# Thangka super-resolution diffusion model based on discrete cosine transform domain padding upsampling and high-frequency focused attention

**DOI:** 10.1371/journal.pone.0332904

**Published:** 2025-09-25

**Authors:** Xin Chen, Liqi Ji, Zhen Wang, Yunbo Yang, Xinyang Zhang, Nianyi Wang

**Affiliations:** 1 Key Laboratory of Linguistic and Cultural Computing, Ministry of Education, Northwest Minzu University, Lanzhou, Gansu, China; 2 School of Mathematics and Computer Science, Northwest Minzu Univsersity, Lanzhou, China; International University of Languages and Media: Libera Universita di Lingue e Comunicazione, ITALY

## Abstract

Thangka is a traditional Tibetan painting art form, possessing profound cultural significance and a unique artistic style. Image super-resolution technology, as an effective means of digital preservation and restoration, plays an important role in maintaining the integrity and heritage of Thangka art. However, existing image super-resolution methods cannot be used for Thangka images due to the following reasons: (1) Thangka images are large in size and rich in content, making it difficult for existing models to restore the original textures of degraded Thangka images. (2) Thangka images have intricate textures, so the high-resolution Thangka images reconstructed by existing methods, which perform well on objective metrics, perform poorly in terms of human visual perception. To overcome these challenges, a Frequency-Domain Enhanced Diffusion Super-Resolution (FDEDiff) is proposed, consisting of three parts: (1) a High-Frequency Focused Cross Attention Mechanism (HFC-Attention), which can separate high-frequency features of images to guide the attention mechanism, improving the reconstruction quality of high-frequency details in the diffusion model; (2) a DCT Domain Padding Upsampling (DCT-Upsampling), which performs upsampling in the Discrete Cosine Transform (DCT) domain and improves the reconstruction of dense line areas in Thangka images by fully utilizing global information; (3) for the first time, we construct a Thangka image super-resolution dataset, which contains 82,688 pairs of 512 × 512 images. Qualitative and quantitative experiments demonstrate that the proposed method achieves state-of-the-art performance on the Thangka dataset, attaining a LPIPS score of 0.0815 (lower indicates better perceptual quality) and showing a 20% improvement in perceptual quality over baseline methods. Although the proposed method requires longer inference time due to the iterative nature of diffusion models, this computational trade-off is justified by the critical need for artistic authenticity in cultural preservation applications. Dataset is available at https://github.com/cvlabdatasets/ThangkaDatasets.

## Introduction

**Fig 1:**
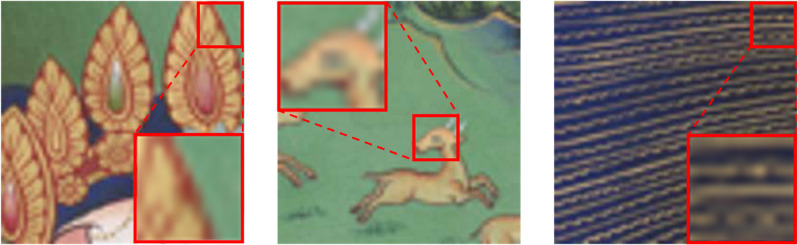
Low-resolution Thangka images. Low-resolution Thangka images do not lack main elements, but the details have significantly degraded. Manual restoration is labor-intensive and inefficient. Using image super-resolution reconstruction techniques to restore Thangka paintings is currently the most efficient and feasible method.

Thangka is a unique form of painting art in Tibetan culture [1], covering a wide range of subjects such as Tibetan history, politics, culture, and social life, characterized by distinctive ethnic features and unique artistic styles. However, due to destruction in history, natural weathering, and the limitations of photographic equipment, some Thangka images have low resolution, posing significant challenges to image preservation (as shown in Fig 1). The effective restoration and preservation of Thangka have become imperative. Advanced digital preservation and restoration technologies have emerged as vital tools in this task. Among these, using image super-resolution reconstruction techniques is a practical method for achieving high-resolution restoration of Thangka images. Super-resolution reconstruction of Thangka can facilitate easier sharing and spreading on the internet and provide researchers with more details for deeper artistic and historical studies, contributing to maintaining global cultural diversity. However, there is currently no research on image super-resolution for Thangka. The rationale for this work stems from the urgent need to develop specialized super-resolution techniques that can effectively preserve and restore Thangka’s unique artistic characteristics, particularly its intricate high-frequency details and densely packed lines, which conventional methods fail to adequately address.

In recent years, with the rapid development of deep learning technology, significant progress has been made in image super-resolution, making the super-resolution reconstruction of Thangka technically feasible. The current main research directions in image super-resolution technology include methods based on Convolutional Neural Networks (CNNs) [2,3], attention mechanisms [4,5], Generative Adversarial Networks (GANs) [6,7], Transformers [8,9], etc. These methods optimize algorithm performance from different perspectives, aiming to improve the visual quality of reconstructed images to meet the demands of practical applications. Despite the excellent results of existing image super-resolution methods, they cannot be directly applied to Thangka image super-resolution due to the following reasons: (1)Thangka images are large in size and rich in content, making it difficult for existing models to restore the original textures of degraded Thangka images. (2)Thangka images have intricate textures, although existing methods excel in metrics based on simple pixel differences, such as peak signal-to-noise ratio (PSNR), they underperform in terms of human visual perception. (3)High-quality training data is critical for image super-resolution, yet no dedicated Thangka dataset exists for this task.

To address the long-standing issue of the lack of datasets suitable for image super-resolution training in Thangka research, we have, for the first time, established a dataset containing 82,688 pairs of 512×512 resolution images, covering various historical styles, and providing a multi-scale evaluation subset. This dataset serves as a reliable benchmark platform for future research.

**Fig 2:**
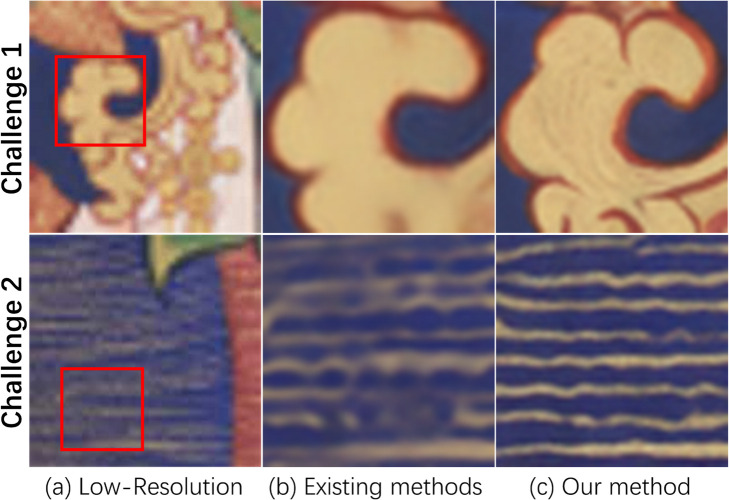
Two challenges faced by existing methods. Existing diffusion model-based methods cannot accurately restore the high-frequency details of Thangka images. The proposed FDEDiff can restore these details more accurately (solution for Challenge1, see Line1). Our method can also more precisely reconstruct the densely packed lines in Thangka images, whereas lines restored by existing models suffer from interruptions, adhesions, and blurriness (solution for Challenge2, see Line2).

Since Ho et al. [10] proposed Denoising Diffusion Probabilistic Models in 2020, diffusion models have become the most advanced deep-learning generative models. Saharia et al. proposed SR3 [11], which demonstrated the tremendous potential of diffusion models in image super-resolution. The core advantage of diffusion models in image super-resolution tasks lies in their progressive detail reconstruction capability and stable training mechanism. Through multi-step iterative denoising, the model restores the overall image structure in the early stages and then gradually refines high-frequency details (such as textures and edges), effectively avoiding the blurring defects of traditional interpolation methods and the over-smoothing issues of CNN-based methods. The training objective directly constrains the noise prediction error at each step, offering a smoother optimization path compared to GANs, which are more prone to mode collapse. However, the application of diffusion model-based super-resolution methods to Thangka images still cannot produce satisfactory results because of two challenges (as shown in Fig 2):

Challenge1: The existing methods based on diffusion model cannot recover the high-frequency details of Thangka images correctly. Due to Thangka’s rich and intricate content, the degraded low-resolution images lack many high-frequency details. The high-frequency details reconstructed by existing methods are random, often resulting in high-frequency details unfaithful to the original image.

Challenge2: Compared with other public data sets, the lines of Thangka images are extremely complex and dense, which makes the diffusion model-based methods unable to correctly identify the residual semantic information in the line-dense areas of low-resolution Thangka images, resulting in the inability to reconstruct the correct lines.

**Fig 3:**
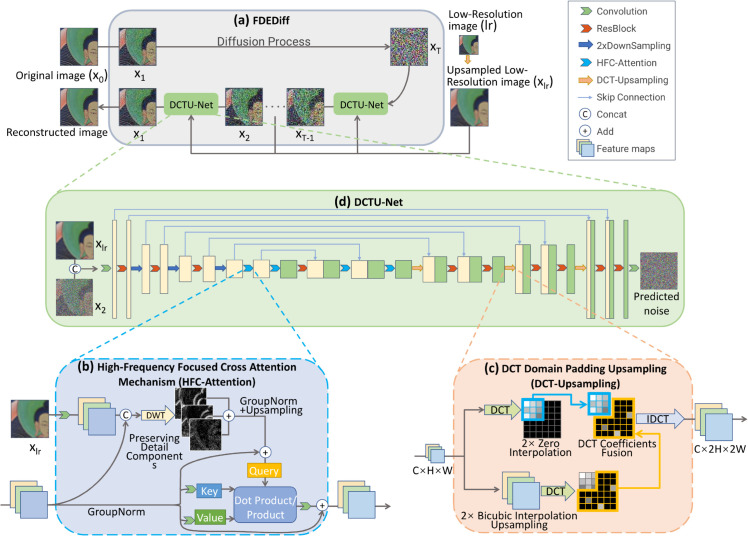
Network architecture of the proposed model. (a) Our method based on HFC-Attention and DCT-Upsampling diffusion model approaches, for learning the high-frequency detail features of Thangka images and fully utilizing global information, and performing super-resolution reconstruction on Thangka images. (b) HFC-Attention, which enhances the model’s focus on high-frequency information, and improves the reconstruction of high-frequency details. (c) DCT-Upsampling, which eliminates the local dependency of the upsampling module, resulting in clearer line textures in the reconstructed images.

**Fig 4:**
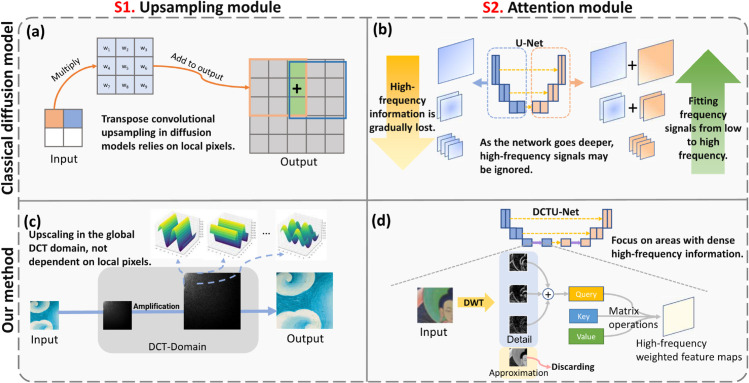
Advantages of the proposed method over classical methods. The sections (a) and (b) in the figure correspond to modules in the classic diffusion model, while (c) and (d) correspond to the proposed method. In column S1, the proposed upsampling method relies entirely on global information, enabling it to better recover degraded textures. In column S2, our method focuses the model on areas dense with high-frequency information, thereby generating more realistic high-frequency details.

The objective of this study is to propose an image super-resolution model specifically tailored for Thangka images, which can effectively restore the unique details characteristic of Thangka art, and to create a high-quality dataset suitable for Thangka image super-resolution tasks. In this paper, a frequency domain enhancement and diffusion model-based solution for Thangka image super-resolution reconstruction is proposed (as shown in Fig 3). The integration of diffusion models and frequency-domain processing stems from their complementary strengths in addressing multiscale restoration challenges. Frequency-domain analysis decomposes images into low-frequency components (global structures) and high-frequency components (local details/noise), enabling targeted enhancement of critical features. This explicit separation overcomes the local receptive field limitations of spatial CNNs. The proposed method consists of two main parts:

(1) First (solution for challenge 1), as shown in Fig 4 S2, we propose a High-Frequency Focused Cross Attention Mechanism (HFC-Attention). This method generates query information for the attention mechanism using high-frequency components obtained through wavelet transform. The high-frequency components of an image contain features such as details and edges. HFC-Attention ensures that the U-Net within the diffusion model focuses on these critical features, thereby accurately generating high-frequency details during image reconstruction. In contrast, the U-Net in existing diffusion models exhibits spectral bias [12,13], preferentially fitting low-frequency signals while neglecting high-frequency ones [14], leading to inaccurate reconstruction of high-frequency details in generated images.

(2) Second (solution for challenge 2), as shown in Fig 4 S1, we propose a DCT Domain [15] Padding Upsampling (DCT-Upsampling). DCT-Upsampling conducts interpolation in the DCT domain and integrates partial high-frequency information from bicubic upsampling. This method enables globally dependent upsampling, avoiding the traditional local pixel dependency of conventional upsampling methods [16] and adds extra high-frequency data. This approach effectively harnesses global information to reconstruct the intricate lines in Thangka.In contrast, the convolutional operations in existing spatial-domain upsampling methods rely on local pixel with limited receptive fields.

Discrete Cosine Transform (DCT) was chosen for the upsampling module due to its energy-compaction property (validated by JPEG standards). Its real-valued computations (vs. Discrete Fourier Transform’s complex operations) ensure compatibility with CNN architectures. Discrete Wavelet Transform (DWT) was adopted for the attention mechanism because its subband decomposition (LH/HL/HH) directly isolates spatially localized high-frequency components—critical for reconstructing Thangka’s dense textures. Unlike Discrete Fourier Transform (DFT), DWT requires no manual cutoff thresholds, preventing accidental loss or contamination of high-frequency details. By jointly encoding what high-frequency elements exist and where they reside, DWT enables precise focus on texture-critical regions during diffusion.

**Fig 5:**
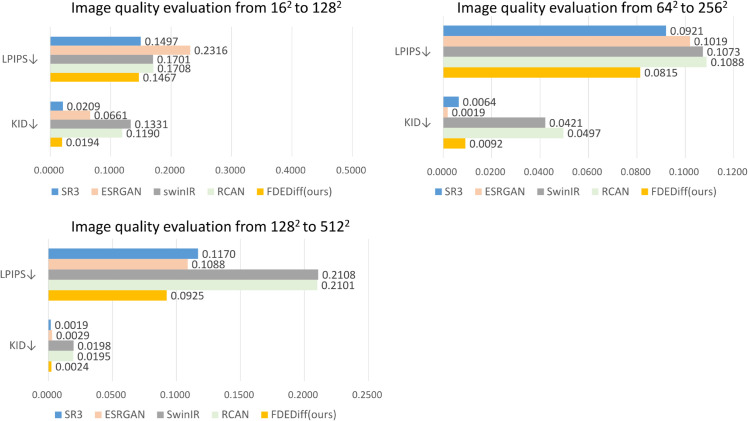
Comparison of image quality metrics. The proposed FDEDiff is compared with existing methods using evaluation metrics LPIPS and KID that reflect human visual perception, which achieves better results on the Thangka dataset.

Super-resolution experiments on Thangka images demonstrate that our method effectively improves the quality of high-frequency details and dense lines in Thangka image super-resolution generation, achieving satisfactory visual performance and favorable evaluation metrics, as shown in Fig 5.

In summary, the main contributions of this work are as follows:

1. We propose a High-Frequency Focused Cross Attention Mechanism (HFC-Attention), which separates high-frequency image features to guide attention, improving the diffusion model’s reconstruction of high-frequency details.
2. We propose a DCT Domain Padding Upsampling (DCT-Upsampling) that performs globally dependent upsampling in the DCT domain, enhancing dense line reconstruction in Thangka images.
3. For the first time, we construct a Thangka image super-resolution dataset containing 82,688 pairs of 512 × 512 images. We hope that this dataset will contribute to research in this area.

## Related work

### Image super-resolution

With the rapid development of digital technology, numerous methods have been proposed to tackle the challenges in image super-resolution. There are three main types of early image super-resolution methods: interpolation-based, reconstruction-based, and shallow learning-based methods [17]. These methods process quickly and can restore the texture details of images to a certain extent. In recent years, image super-resolution has successfully integrated deep learning techniques, making deep learning-based image super-resolution methods the mainstream [18]. Dong et al. [2] pioneered a model based on CNNs for image super-resolution, which marked a revolutionary innovation. Subsequently, researchers have proposed many improvements and innovations, with later models effectively enhancing the quality and efficiency of image super-resolution through deeper network structures, more enhanced feature extraction capabilities, and more complex learning strategies. Techniques such as residual learning (VDSR [19], EDSR [20]) and dense connections (SRDenseNet [21]) reduce information loss during training, which improves the quality of image super-resolution. Furthermore, some studies have proposed attention mechanisms [4] [5] [22] [23] [24] [25] [26], Transformer architectures and GANs [6] [27] [28] [29]. By focusing on crucial feature areas of the image, attention mechanisms improve the details and textures, thereby enhancing perceptual quality. GANs through the adversarial process between generators and discriminators, promote more realistic image reconstruction, enhancing the realism of images.

However, these methods all have their inherent limitations: Traditional interpolation-based, reconstruction-based, and shallow learning-based methods are generally limited by lower reconstruction quality and detail recovery capability, especially in handling high magnification and complex textures, often failing to effectively reconstruct lost high-frequency details, leading to blurred and distorted images. Residual learning and dense connections often come with higher computational and memory costs and potential overfitting and training stability issues. Although effective at highlighting key features, super-resolution models based on attention mechanisms have problems with high computational complexity, difficulties in generalizing to different types of images, and efficiency issues in processing global information. GANs, although capable of generating high-quality images, often have problems with training instability and the propensity to produce artifacts.

### Diffusion model-based image super-resolution reconstruction methods

Diffusion models generate high-quality, diverse images, especially regarding the authenticity and creativity of details. Diffusion models gradually construct complex images through a step-by-step refinement process. With their exceptional ability to capture and reproduce highly complex and detailed image distributions, diffusion models have significantly enhanced the performance and quality of image generation tasks compared to previous generative models [30]. Li et al. [31] proposed a T-step diffusion model for single-image super-resolution, emphasizing the use of conditional noise predictors and an LR encoder to predict differences between high-resolution and low-resolution images. Saharia et al. proposed SR3 [11], a super-resolution diffusion model, excels at converting low-resolution images into high-resolution versions starting from pure noise, demonstrating strong performance in super-resolution tasks for faces and natural images. Shang et al. proposed ResDiff [32], which combines convolutional neural networks (CNNs) with diffusion probabilistic models for single-image super-resolution. ResDiff uses CNNs to restore the main low-frequency components, while the diffusion model predicts the residual between the CNN-predicted image and the true high-resolution image, thereby accelerating the generation process and producing higher-quality samples. Wang et al. proposed a novel approach for blind super-resolution by leveraging prior knowledge from a pre-trained text-to-image diffusion model [33]. By using a time-aware encoder without altering the pre-trained generative model, they achieved satisfactory restoration results.

However, the transpose convolution upsampling used in the U-Net within diffusion models relies on local information, leading to a lack of global dependency in feature maps during processes such as upsampling [34]. And, the U-Net in diffusion models exhibits spectral bias [12,13], preferring to fit low-frequency signals and tending to ignore high-frequency signals [14], which results in inaccurate high-frequency details in the generated images. Despite significant progress in diffusion models across multiple domains, current research primarily focuses on multimodal applications. In contrast, studies related to diffusion models focusing on image super-resolution reconstruction are relatively limited.

### Frequency domain learning

Frequency domain learning focuses on processing and understanding data within the frequency domain, effectively revealing global patterns and high-frequency information in images. This approach is particularly suitable for handling image data, as it can efficiently capture and analyze global features across different frequencies. Rippel et al. [35] first proposed the concepts of spectral pooling and spectral parametrization, validating the effectiveness of learning convolutional neural network filters directly in the frequency domain, which significantly improved information preservation and network training speed. Xu et al. [36] proposed a frequency domain learning method that reshapes images in the frequency domain and uses DCT coefficients as input to optimize the performance of image processing tasks. Chi et al. [37] proposed a fast Fourier convolution operator to replace traditional convolution, successfully enhancing the performance of visual tasks. Cai et al. [38] proposed FreqNet, which converts images to the frequency domain through discrete cosine transform, focusing on learning high-frequency information to improve the effects of single-image super-resolution networks. Wang et al. [39] enhanced the performance of GANs training on limited data by focusing on the high-frequency components of images. Yu et al. [16] effectively overcame the limitations of traditional spatial upsampling operations, achieving performance improvements in multiple visual tasks by upsampling in the Fourier domain.

However, current research on frequency domain learning methods is primarily conducted on traditional models such as CNNs and GANs, and integration with existing advanced models, such as diffusion models, remains underexplored.

## Methodology

As shown in Fig 3, the proposed Frequency-Domain Enhanced Diffusion Super-Resolution (FDEDiff) method for Thangka image restoration employs DCTU-Net as its core denoising network. The framework comprises two key innovations: (1) A High-Frequency Focused Cross Attention Mechanism (HFC-Attention) to enhance the model’s attention to high-frequency details and improve the reconstruction of image high-frequency details; (2) A DCT Domain Padding Upsampling (DCT-Upsampling), which’s a globally dependent upsampling method that introduces additional high-frequency signals, aiding in the improvement of dense line area reconstruction in Thangka images. Our method effectively enhances the super-resolution reconstruction quality of Thangka images.

This chapter elaborates on the methodology through three perspectives: (a) architectural overview of the diffusion framework, (b) operational principles of the denoising diffusion model, and (c) implementation details of the proposed HFC-Attention and DCT-Upsampling modules.

### Overview of network architecture

As illustrated in Fig 3(a), FDEDiff initiates with a pure Gaussian noise image that iteratively converges to high-resolution outputs under the guidance of low-resolution references. As visualized in Fig 3(d), the DCTU-Net predicts noise residuals at each diffusion step through spatial-frequency co-optimization — a hybrid strategy where spatial convolutions capture local textural patterns while DCT-domain operations enforce global structural coherence. The DCTU-Net features a symmetric encoder-decoder architecture. The encoder path comprises four-stage hierarchical feature extraction modules, each progressively capturing Thangka image features through three ResNet blocks. Shallow layers employ dilated convolutions to extract local texture details, while our novel high-frequency focused cross-attention mechanism explicitly enhances the modeling capability for both high-frequency patterns (e.g., hair strands and plant venations) and semantic structures (e.g., deity postures and ritual instrument arrangements) in Thangka images.

The decoder path innovatively integrates a configurable frequency-domain interpolation module (defaulting to DCT-domain zero-padding strategy), which performs globally dependent upsampling by transforming feature maps into the DCT domain during decoding. Multi-scale feature reconstruction is achieved through skip connections that preserve granular texture information. The bottleneck layer between encoder and decoder adopts a dual-residual structure: the first residual block enhances spatial continuity via global self-attention, while the second suppresses noise interference through channel rearrangement, forming a “local-global-local" feature refinement loop.

DCTU-Net implements a guided conditioning strategy by concatenating low-resolution inputs with timestep embeddings to steer the generation process, ensuring semantic consistency of religious symbols and textural details unique to Thangka iconography. These architectural innovations preserve the generative advantages of diffusion models while substantially mitigating line discontinuity and high-frequency distortion prevalent in conventional methods, with detailed implementation mechanisms elaborated in subsequent sections.

### Denoising diffusion model

Diffusion models approach image generation as a gradual denoising process governed by two complementary phases: a forward diffusion process that incrementally corrupts training images with Gaussian noise, and a learned reverse diffusion process that iteratively recovers clean images from noise. In the context of Thangka super-resolution, this framework is adapted as a conditional generation task — starting from pure noise, the model progressively synthesizes high-frequency details (e.g., intricate deity ornaments) and structural patterns (e.g., geometrically aligned mandalas) under the guidance of low-resolution inputs. Each denoising step combines: (1) Noise prediction via a U-Net trained to estimate the perturbation added at specific timesteps, (2) Conditional refinement where LR features steer the reconstruction toward iconographically valid outputs, (3) Frequency-domain constraints through our novel modules to preserve textural authenticity. Unlike GANs that directly map LR to HR domains, this iterative paradigm enables controlled detail hallucination while maintaining spatial coherence critical for art preservation.

#### Forward diffusion process.

Given an initial data distribution ${𝐱}_{0}\sim q(𝐱)$, Gaussian noise is continuously added to the distribution, sampling a series of noisy samples ${𝐱}_{1},\ldots ,{𝐱}_{T}$, with the variance of this Gaussian noise determined by fixed values $\{\beta_{t}\in (0,1){\}}_{t=1}^{t}$, and the mean determined by the fixed value $\beta_{t}$ and the data ${𝐱}_{t}$ at the current time *t*. This process is a Markov chain process, meaning the probability of the next state depends only on the current state and not on previous historical states. As *t* increases, the final data distribution ${𝐱}_{T}$ approaches an isotropic Gaussian distribution.

$$q({𝐱}_{t}|{𝐱}_{t-1})=𝒩({𝐱}_{t};\sqrt{1-\beta_{t}}{𝐱}_{t-1},\beta_{t}𝐈)$$ (1)

$$q({𝐱}_{1:T}|{𝐱}_{0})=\prod_{t=1}^{T}q({𝐱}_{t}|{𝐱}_{t-1})$$ (2)

where $q({𝐱}_{t}|{𝐱}_{t-1})$ denotes the probability distribution of state ${𝐱}_{t}$ given the previous state ${𝐱}_{t-1}$. This distribution is modeled as a Gaussian distribution with a mean of $\sqrt{1-\beta_{t}}{𝐱}_{t-1}$ and variance $\beta_{t}𝐈$, where $\beta_{t}$ is a predefined parameter and *I* represents the identity matrix, indicating that the covariance matrix is diagonal. $q({𝐱}_{1:T}|{𝐱}_{0})$ represents the joint probability distribution of the entire sequence from initial state ${𝐱}_{0}$ to state ${𝐱}_{T}$, computed through the product $\prod_{t=1}^{T}q({𝐱}_{t}|{𝐱}_{t-1})$. This formulation reveals the dynamic evolution of the state sequence, where each state is dependent on its immediate predecessor. The distribution $q({𝐱}_{t})$ at any time can be fully derived based on ${𝐱}_{0}$ and $\beta_{t}$ without iteration. Let $\alpha_{t}=1-\beta_{t}$ and ${\bar{\alpha }}_{t}=\prod_{i=1}^{t}\alpha_{i}$. Through derivation, we obtain:

$${𝐱}_{t}=\sqrt{{\bar{\alpha }}_{t}}{𝐱}_{0}+\sqrt{1-{\bar{\alpha }}_{t}}\epsilon ,\;\epsilon \sim 𝒩(0,𝐈)$$ (3)

where $\alpha_{t}$ dictates the influence of the initial state on the current state ${𝐱}_{t}$, while $\sqrt{1-\alpha_{t}}\epsilon$ adds a random noise component. This noise, *ε*, is drawn from a standard normal distribution *N*(0,*I*), introducing an element of uncertainty into the state evolution.

$$q({𝐱}_{t}|{𝐱}_{0})=𝒩({𝐱}_{t};\sqrt{{\bar{\alpha }}_{t}}{𝐱}_{0},(1-{\bar{\alpha }}_{t})𝐈)$$ (4)

Based on Eq 3, Eq 4 is derived by considering the distribution of the noise term *ε*, which is standard normal, leading to a Gaussian distribution for ${𝐱}_{t}$ with the mean and variance specified.

#### Reverse diffusion process.

Assuming we can estimate $q({𝐱}_{t-1}|{𝐱}_{t})$, we can reverse the above diffusion process and sample from $q({𝐱}_{t-1}|{𝐱}_{t})$, thus obtaining real samples ${𝐱}_{1},\ldots ,{𝐱}_{T}$ from the Gaussian noise input ${𝐱}_{T}\sim 𝒩(0,𝐈)$. The reverse diffusion process recovers original data from Gaussian noise. We assume it is also a Gaussian distribution. However, it is not feasible to fit the distribution $q({𝐱}_{t-1}|{𝐱}_{t})$ step by step, so a parameterized distribution is constructed for estimation. Drawing from the theoretical explanation of Variational Autoencoders (VAE) [40], diffusion models belong to Likelihood-based Models. Thus, the optimization goal is to maximize the likelihood estimate of the true data distribution $p_{\theta }({𝐱}_{0})$, where *θ* represents the parameters learned by a neural network.

$$p_{\theta }({𝐱}_{0:T})=p({𝐱}_{T})\prod_{t=1}^{T}p_{\theta }({𝐱}_{t-1}|{𝐱}_{t})$$ (5)

where the joint probability distribution of the entire sequence is represented, which can be seen as a reverse diffusion process from the final state ${𝐱}_{T}$ to the initial state ${𝐱}_{0}$.

$$p_{\theta }({𝐱}_{t-1}|{𝐱}_{t})=𝒩({𝐱}_{t-1};\mu_{\theta }({𝐱}_{t},t),\Sigma_{\theta }({𝐱}_{t},t))$$ (6)

where the conditional probability distribution of the previous state ${𝐱}_{t-1}$, given the subsequent state ${𝐱}_{t}$, is specified. This is a Gaussian distribution, where the mean and covariance parameters are output by a neural network. By performing a series of derivations, we derive the posterior distribution of ${𝐱}_{t-1}$ given $\left({𝐱}_{0},{𝐱}_{t}\right)$.

$$q({𝐱}_{t-1}|{𝐱}_{t},{𝐱}_{0})=𝒩({𝐱}_{t-1};\tilde{\mu }({𝐱}_{t},{𝐱}_{0}),{\tilde{\beta }}_{t}𝐈)$$ (7)

where ${\tilde{\beta }}_{t}$ and ${\tilde{\mu }}_{t}({𝐱}_{t},{𝐱}_{0})$ are calculated as follows:

$${\tilde{\beta }}_{t}=\frac{1-{\bar{\alpha }}_{t-1}}{1-{\bar{\alpha }}_{t}}\cdot \beta_{t}$$ (8)

$${\tilde{\mu }}_{t}({𝐱}_{t},{𝐱}_{0})=\frac{\sqrt{\alpha_{t}}\left(1-{\bar{\alpha }}_{t-1}\right)}{1-{\bar{\alpha }}_{t}}{𝐱}_{t}+\frac{\sqrt{{\bar{\alpha }}_{t-1}}\beta_{t}}{1-{\bar{\alpha }}_{t}}{𝐱}_{0}$$ (9)

To maximize the likelihood estimate of the true data distribution $p_{\theta }({𝐱}_{0})$, the optimization goal, after a series of derivations, can be expressed as

$$\underset{\theta }{*\arg\min}D_{KL}\left(q\left(x_{t-1}|x_{t},x_{0}\right)\|p_{\theta }\left(x_{t-1}|x_{t}\right)\right)=\underset{\theta }{*\arg\min}\frac{1}{2\sigma_{q}^{2}\left(t\right)}\frac{{\left(1-\alpha_{t}\right)}^{2}}{\left(1-{\bar{\alpha }}_{t}\right)\alpha_{t}}\left[{\left\|\varepsilon_{\theta }(x_{t},t)-\varepsilon_{t}\right\|}_{2}^{2}\right]$$ (10)

where $\varepsilon_{\theta }$ represents the noise term predicted by the model parameters *θ*, while $\varepsilon_{t}$ refers to the noise added during the diffusion process at time step *t*. $\sigma_{q}^{2}(t)$ denotes the variance of the noise in the distribution $q(x_{t-1}|x_{t},x_{0})$ at time *t*, which impacts the estimation of the KL divergence [41]. This leads to a simplified loss function

$$L_{simple}(\theta )={𝔼}_{t,{𝐱}_{0},\epsilon }[{\left\|\epsilon -\epsilon_{\theta }(\sqrt{{\bar{\alpha }}_{t}}{𝐱}_{0}+\sqrt{1-{\bar{\alpha }}_{t}}\epsilon ,t)\right\|}^{2}]$$ (11)

where $\epsilon_{\theta }$ represents the noise predicted by the model *θ*, and ϵ represents the standard Gaussian noise added during the diffusion process.

#### Conditional generation with diffusion models.

**Algorithm 1** The T-step iterative denoising algorithm.

$𝐱=DCT-Upsampling({𝐱}_{lr})$

${𝐱}_{T}\sim 𝒩(0,𝐈)$

**for** t=T,…,1 **do**

ϵ~𝒩(0,𝐈) if *t*>1, otherwise ϵ=0

${𝐱}_{t-1}=\frac{1}{\sqrt{\alpha_{t}}}\left({𝐱}_{t}-\frac{1-\alpha_{t}}{\sqrt{1-{\bar{\alpha }}_{t}}}\epsilon_{\theta }\left(𝐱,{𝐱}_{t},t\right)\right)+\sqrt{1-\alpha_{t}}\epsilon$

**end for**

Return ${𝐱}_{0}$

We train a denoising model, *θ*, which infers $\epsilon_{\theta }$ to fit the noise *ε* added at each step of the forward process. DCT-Upsampling receives the source image ${𝐱}_{lr}$ and outputs **x** as additional information. **x**, the time step *t*, and the noisy target image ${𝐱}_{t}$ together serve as inputs to $\epsilon_{\theta }$, which is trained to predict the noise vector *ε*. According to Equation (2), given ${𝐱}_{0}$ and $\beta_{t}$, we can derive $q({𝐱}_{t})$.

We aim to recover the noise-free target image ${𝐱}_{0}$ by predicting the noise vector *ε*. The T-step iterative denoising algorithm of the diffusion models is shown as Algorithm 1.

### High-frequency focused cross attention mechanism (HFC-attention)

In diffusion models, the U-Net, during the downsampling process, increases the number of channels to maintain or enhance the network’s information capacity. However, the reduction of spatial information often leads to the loss of some detail information. Our High-Frequency Focused Cross Attention Mechanism (HFC-Attention) module specifically targets areas rich in high-frequency details, enhancing the U-Net’s ability to represent high-frequency information and thereby improving the quality of high-frequency details in diffusion model super-resolution reconstruction. Although traditional attention mechanisms can enhance the representational power of features, they often do not specifically focus on high-frequency information.

The proposed HFC-Attention module consists of three core components: (1) cross-level feature fusion, (2) high-frequency component extraction, and (3) frequency-guided attention computation. As shown in Fig 3(c), this module is deployed at critical connection points between the U-Net encoder and decoder.

#### Cross-level feature fusion.

The module receives two inputs: the low-resolution guidance image $X\in {\mathbb{R}}^{3\;\times \;H\;\times \;W}$ and intermediate feature map $F_{input}\in {\mathbb{R}}^{C\;\times \;H\;\times \;W}$. Feature fusion is achieved through concatenation and 1×1 convolution:

$$F_{matched}={\text{Conv}}_{1\;\times \;1}(\text{Concat}(F_{input},{\text{Conv}}_{1\;\times \;1}(X)))$$ (12)

This integration provides pixel-wise structural guidance from the LR image, anchoring the generation process to preserve output content fidelity.

#### High-frequency component extraction.

We employ Haar basis-based Discrete Wavelet Transform (DWT) to decompose the fused features:

$$F_{matched}={\text{Conv}}_{1\;\times \;1}(\text{Concat}(F_{input},{\text{Conv}}_{1\;\times \;1}(X)))$$ (13)

where *F*_*LL*_ contains low-frequency approximation components, while *F*_*LH*_, *F*_*HL*_, and *F*_*HH*_ capture horizontal, vertical, and diagonal high-frequency details respectively. The high-frequency components are aggregated as:

$$F_{high\_frequency}=[F_{LH},F_{HL},F_{HH}]$$ (14)

This design leverages the multi-directional decomposition characteristics of Haar wavelets. Although various wavelet bases exist, Haar wavelet is selected for its computational efficiency and suitability for capturing image abrupt transitions (e.g., clothing decorative stripes, animal fur). Ayas and Ekinci [42] demonstrated through comparative experiments that although wavelets such as Daubechies db4 perform well in certain cases, the Haar wavelet achieves the best trade-off between computational efficiency and reconstruction quality. Unlike Fourier transform’s global frequency representation, Haar wavelet transform achieves joint spatial-frequency analysis through time-frequency localized basis functions. This property enables precise capture of Thangka textures’ non-stationary characteristics - i.e., the localized spatial concentration of high-frequency details. As shown in Fig 4(d), the horizontal (*F*_*LH*_), vertical (*F*_*HL*_), and diagonal (*F*_*HH*_) subbands from wavelet decomposition correspond to edge transitions in different orientations, providing direction-sensitive detail priors for subsequent attention mechanisms.

#### Frequency-guided attention computation.

The attention mechanism couples with high-frequency features through a three-stage process. First, query vector construction:

$$Q={\text{Conv}}_{1\;\times \;1}(\text{GroupNorm}(\text{Upsampling}(F_{high\_frequency})+F_{input}))$$ (15)

By summing upsampled high-frequency features $F_{high\_frequency}$ with original features *F*_*input*_, the query vectors are forced to carry high-frequency localization information.

Next, key-value pair generation:

$$K,V={\text{Conv}}_{1\;\times \;1}(\text{GroupNorm}(F_{input}))$$ (16)

Here, Group Normalization (GroupNorm) is applied to the original features to preserve local structural continuity while suppressing inter-channel covariate shift.

Finally, attention fusion with residual connection:

$$F_{output}={\text{Conv}}_{1\;\times \;1}(\text{Softmax}(\frac{QK^{T}}{\sqrt{d_{k}}})V)+F_{input}$$ (17)

Through high-frequency guided attention weights, the model autonomously focuses on texture transition regions. The residual connection ensures enhanced high-frequency details without compromising the U-Net’s inherent semantic encoding capability.

By explicitly injecting high-frequency features into query vectors, HFC-Attention compels the attention mechanism to prioritize texture transition regions. This strategy effectively overcomes the spectral bias in traditional diffusion model U-Nets - i.e., excessive fitting of low-frequency signals while neglecting high-frequency details - achieving a 5% LPIPS improvement in complex line reconstruction (see ablation studies in Table 5).

### DCT domain padding upsampling (DCT-Upsampling)

**Fig 6:**
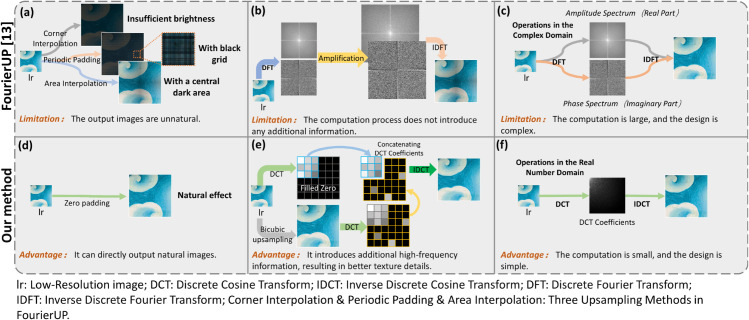
Advantages of DCT-Upsampling. Compared with FourierUP [16], the proposed FDEDiff has significant advantages. The images produced by FourierUP [16] are less natural (as seen in d) than those produced by our method (as seen in a). Compared to our method (as seen in e), FourierUP [16] does not introduce any additional high-frequency information, while our method achieves better texture details (as seen in b). FourierUP [16] requires calculations in the complex domain (as seen in c), while our method performs calculations in the real domain, resulting in lower computational cost (as seen in f).

Compared to the spatial domain, the Fourier domain does not possess the same scale invariance properties and local texture similarity. Thus, the same techniques used in the spatial domain cannot be applied for upsampling. Inspired by FourierUp [16], we propose the DCT-Upsampling method as shown in Fig 3(c). This approach achieves global image magnification through DCT domain upsampling, eliminating dependence on local pixels. The DCT-Upsampling module implements a hybrid magnification strategy through three synergistic operations: (1) global DCT domain padding, (2) spatial domain high-frequency enhancement, and (3) adaptive frequency-domain fusion. As illustrated in Fig 6(d, e, f), this design combines the global consistency of DCT domain processing with the local adaptability of spatial operations.

#### Global DCT domain padding.

Given a low-resolution input $I_{lr}\in {\mathbb{R}}^{C\;\times \;H\;\times \;W}$, we first transform it to the DCT domain:

$$F_{DCT\_lr}^{\left[C\;\times \;H\;\times \;W\right]}=\text{DCT}(I_{lr})$$ (18)

where $F_{DCT\_lr}^{\left[C\;\times \;H\;\times \;W\right]}$ denotes the DCT coefficient matrix of size H×W. The zero-padding operation in DCT domain can be mathematically formulated as:

$$F_{DCT\_ZeroPadding}^{\left[C\;\times \;2H\;\times \;2W\right]}(u,v)=\left\{ \begin{array}{cc} F_{DCT\_lr}(u,v), & 0\leq u<H,\;0\leq v<W\\ 0, & \text{otherwise} \end{array}\right.$$ (19)

ensuring complete preservation of original signal energy while doubling spatial resolution.

#### Spatial domain high-frequency enhancement.

Concurrently with DCT domain processing, we perform bicubic upsampling in the spatial domain:

$$I_{BicubicUpsampling}=\text{BicubicUpsampling}(I_{lr})\in {\mathbb{R}}^{C\;\times \;2H\;\times \;2W}$$ (20)

The upsampled image is then transformed to the DCT domain:

$$F_{DCT\_BicubicUpsampling}=\text{DCT}(I_{BicubicUpsampling})$$ (21)

This branch compensates for high-frequency vacancies in pure DCT domain padding methods, as the high-frequency regions in the “Global DCT Domain Padding" spectrum remain zero-initialized.

#### Adaptive frequency-domain fusion.

High-frequency component fusion is achieved through a two-stage strategy. First, a frequency band selection scheme defines the boundary between low- and high-frequency components:

$$M(u,v)=\left\{ \begin{array}{cc} 0, & \text{if }u<\alpha H\text{ and }v<\alpha W\;(\text{retain low-frequency core})\\ 1, & \text{otherwise}\;(\text{inject high-frequency details}) \end{array}\right.$$ (22)

where *M*(*u*,*v*) serves as the DCT coefficient mask for frequency separation, with *α* controlling the high-frequency preservation range (default: 0.5).

Next, a hybrid enhancement strategy adaptively fuses the global DCT interpolation’s low-frequency structure with the spatial bicubic upsampling’s high-frequency details through mask-controlled spectrum blending:

$$F_{DCT\_enhanced}=\underset{\text{global structure}}{\underbrace{F_{DCT\_ZeroPadding}}}+\underset{\text{partial details}}{\underbrace{F_{DCT\_up}}}⊙M$$ (23)

This design comprehensively preserves the low-frequency geometric structures of Thangka patterns (via the top-left quadrant of $F_{DCT\_ZeroPadding}$) while avoiding symbolic distortion risks. Moreover, the high-frequency components injected through the spatial branch (mask *M* regions) primarily originate from spatial domain interpolation predictions, effectively compensating for detail reconstruction deficiencies in pure frequency-domain methods (as DCT zero-padding introduces no additional high-frequency signals).

#### Advantages over FourierUp.

FourierUp [16] pioneers frequency-domain upsampling by leveraging Discrete Fourier Transform (DFT) to achieve global dependency modeling. Compared to Fourier transform-based FourierUp [16], our method exhibits three distinct advantages:

**Artifact-free output**: Yu et al. proposed three FourierUp variants [16] (Periodic Padding, Area Interpolation-Cropping, and Corner Interpolation). These methods produce amplified images with defects like insufficient brightness, dark grids, and central shadows (Fig 6 a), requiring convolutional neural network correction before downstream processing. Our DCT-Upsampling maintains DCT domain global dependencies while directly producing usable amplified images (Fig 6 d).

**High-frequency enhancement**: FourierUp variants [16] introduce limited additional information through specific interpolation methods (Fig 6 b). DCT-Upsampling supplements high-frequency information generated by bicubic upsampling (Fig 6 e), optimizing output clarity and facilitating dense line texture reconstruction.

**Real-domain efficiency**: FourierUp [16] operates in the Discrete Fourier Transform (DFT [43]) domain, requiring complex number computations for both amplitude and phase (Fig 6 c). DCT-Upsampling performs upsampling in the real-number DCT domain, eliminating complex arithmetic while achieving comparable or superior results with reduced computational load (Fig 6 f).

## Experimental results and discussion

### Experimental details

The proposed model was trained on a Thangka dataset that we created ourselves. The training was divided into three groups: $16^{2}\to 128^{2}$, $64^{2}\to 256^{2}$, and $128^{2}\to 256^{2}$. We used Adam as the optimizer with specific hyperparameters for each task shown in Table 1. The diffusion process employed a linear noise schedule with 2,000 timesteps, where the noise variance linearly increases from $1\;\times \;10^{-6}$ to $1\;\times \;10^{-2}$. To enhance training stability and generalization, we applied standard data augmentation techniques including random horizontal/vertical flips and random rotations (90^°^). Exponential moving average (EMA) with a decay rate of 0.9999 was used to stabilize the training process. We first preprocess the high-resolution Thangka image *I*_*hr*_ by downsampling it to a smaller size, obtaining *I*_*lr*_. Then, using our DCT-Upsampling method, *I*_*lr*_ is upsampled to the same size as *I*_*hr*_, resulting in the image **x**. **x** is used as a low-resolution guide image input into the model, serving as the condition for image generation, and finally, the reconstructed super-resolution image ${𝐱}_{0}$ is output. Each group of experiments was conducted on a single NVIDIA 3090 RTX GPU for 1000,000 iterations of training, which yielded promising and advantageous results.s of training, which yielded promising and advantageous results.

**Table 1:** Learning rate settings for super-resolution tasks.

Task	Learning rate
$16^{2}\to 128^{2}$	0.0001
$64^{2}\to 256^{2}$	0.000003
$128^{2}\to 512^{2}$	0.000001

Table outlines the specific learning rates applied to different super-resolution scaling tasks within our experimental setup.

### Qualitative evaluation

**Fig 7:**
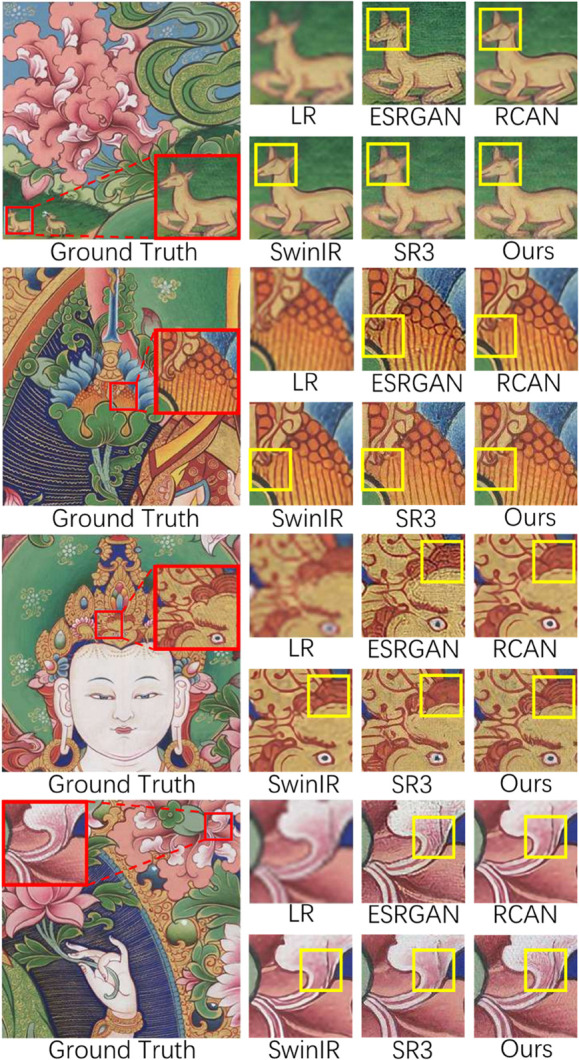
Qualitative evaluation results. Comparison with state-of-the-art image super-resolution methods. Compared to RCAN [4], SwinIR [8], ESRGAN [6], and SR3 [11], our method achieves better visual effects in restoring high-frequency details and reconstructing dense lines. The results of RCAN and SwinIR are too smooth, ESRGAN’s results contain artifacts, and SR3’s results have unclear lines.

In Fig 7, we compare the proposed method with several state-of-the-art image super-resolution reconstruction methods in recent years, including RCAN [4], SwinIR [8], ESRGAN [6], and SR3 [11]. As shown in the second row of Fig 7, the reconstruction result of RCAN presents unnatural uniform color blocks in the Thangka lotus pattern area, with blurred line edges. This is mainly attributed to the characteristics of the residual channel attention mechanism and the high-frequency suppression effect. RCAN dynamically adjusts feature weights through channel attention, but the deep residual structure (more than 400 layers) causes low-frequency information to be repeatedly reinforced in multiple residual connections [4]. On the other hand, channel attention tends to enlarge global statistical features (such as color distribution), while high-frequency details of Thangka images (such as line edges) are suppressed due to their higher local variance [5].

SwinIR generates misaligned lines in areas with dense textures, such as the clothing patterns of Thangka Buddha figures (see Fig 7, second row). This occurs because the relative position encoding used by SwinIR models local positional relationships through predefined offsets but is sensitive to global structure, dynamic deformations, and cross-window dependencies. When the texture exceeds its local modeling capability or introduces positional ambiguities, misalignment in the reconstruction is likely, especially when dealing with the rich high-frequency repetitive patterns (e.g., checkerboards, dense stripes, or grids), symmetric structures, and long-range periodic textures (such as regular grids) in Thangka images.

As shown in the second and third rows of Fig 7, ESRGAN generates artifacts in areas with dense lines. This is likely due to its adversarial training mechanism, which is prone to mode collapse under the strong regularity of Thangka textures (referencing studies on the limitations of GANs in structured textures).

As shown in Fig 7, the local line distortions and texture blurring in SR3 confirm the spectral bias issue of the diffusion model U-Net—its preference for low frequencies leads to inadequate fitting of high-frequency edge signals [14].

**Fig 8:**
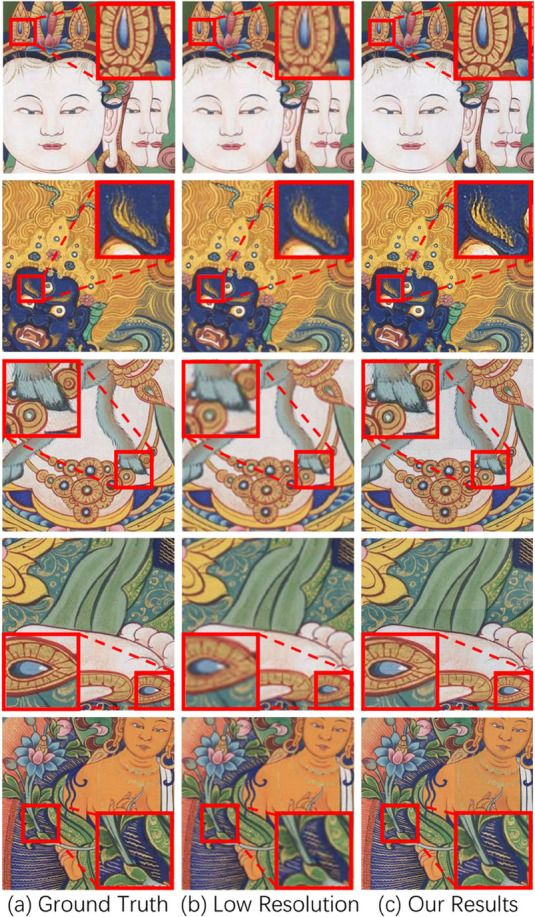
More cases of Thangka image super-resolution using our method.

**Fig 9:**
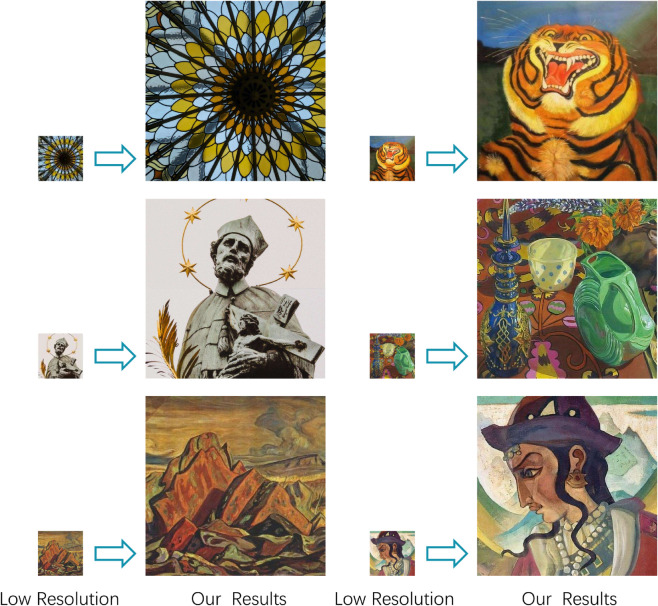
Super-resolution results of our method on public datasets (DIV2K [44], WikiArt [45]).

Compared to the aforementioned image super-resolution reconstruction methods, our method significantly enhances the accuracy of high-frequency detail reconstruction, achieving accurate restoration of dense line textures and improving the super-resolution reconstruction effect of Thangka images. Our HFC-Attention explicitly separates high-frequency components as attention queries through wavelet decomposition, forcing the model to prioritize the rich details unique to Thangka images (see Fig 8, 2nd and 3rd rows). This fundamentally differentiates our approach from RCAN/SwinIR, which rely solely on spatial domain attention. DCT-Upsampling preserves the integrity of edge structures, such as lines, through global interpolation in the frequency domain (see Fig 8, 1st, 4th, and 5th rows). There are more image super-resolution results on Thangka images using our method (Fig 8), along with results from some public datasets (Fig 9).

### Quantitative evaluation

To thoroughly evaluate the performance of the proposed model, we utilized five assessment metrics: Peak Signal-to-Noise Ratio (PSNR), Structural Similarity Index (SSIM [46]), Fréchet Inception Distance (FID [47]), Kernel Inception Distance (KID [48]), and Learned Perceptual Image Patch Similarity (LPIPS [49]). These metrics allow us to compare our method with cutting-edge image super-resolution reconstruction techniques. PSNR measures image quality based on the maximum possible pixel value of the image and the error between images. A higher PSNR generally indicates a smaller error between the reconstructed image and the original image. SSIM is a model used to measure the similarity between two images. The closer the SSIM value is to 1, the more similar the images are; values closer to 0 or negative values indicate greater differences. FID measures the difference in the feature distribution between generated and real images. A lower FID value indicates more similar distributions, implying higher quality of generated images. FID values are non-negative, with closer to 0 being better. KID measures the expected distance in the embedding space between generated and real images. Similar to FID, lower KID values indicate higher image quality, but KID is more statistically stable in significance testing. LPIPS assesses the perceptual difference between images, using features extracted by deep networks to calculate similarity. The smaller the LPIPS, the better the perceptual quality and the more similar the images.

We evaluated the super-resolution reconstruction performance of Thangka images on Thangka dataset, Manga109 [50], and Set5 [51]. The average values were taken for several metrics including PSNR, SSIM and LPIPS. Due to their characteristics, FID and KID do not require averaging. As shown in Table 2, 3, and 4, in three sets of super-resolution reconstruction experiments: $16^{2}\to 128^{2}$, $64^{2}\to 256^{2}$, and $128^{2}\to 256^{2}$, the proposed model achieved top-2 results in FID, KID, and LPIPS metrics on the Thangka dataset, demonstrating superior visual effect in generated images. This advantage is sustained across Manga109 [50] and Set5 [51] datasets, demonstrating the generalizability of the approach to diverse image content. Notably, CNN and Transformer-based methods (RCAN [4], SwinIR [8]) achieve superior performance compared to FDEDiff in terms of PSNR and SSIM metrics. RCAN [4]/SwinIR [8] leverage deep convolutional layers and window attention mechanisms to reconstruct local structures, demonstrating advantages in pixel-level accuracy. However, FDEDiff exhibits better performance in visual realism metrics (FID/KID/LPIPS). This is attributed to FDEDiff’s tendency to generate plausible rather than pixel-perfect reconstructions. On the other hand, compared to other generative models (ESRGAN [6], SR3 [11]), FDEDiff demonstrates clear advantages in PSNR/SSIM metrics. This improvement stems from FDEDiff’s reduced artifacts and hallucinations compared to ESRGAN [6] and SR3 [11].

**Table 2:** Image quality evaluation from $16^{2}\to 128^{2}$.

Method	FID↓	KID↓	LPIPS↓	PSNR↑/ SSIM↑
Thangka Dataset				
FDEDiff(ours)	**108.7645**	**0.0194**	**0.1467**	**22.4200/0.5958**
RCAN [4]	210.1504	0.1190	0.1708	24.4090/0.6572
SwinIR [8]	233.2038	0.1331	0.1701	24.5730/0.6639
ESRGAN [6]	154.2661	0.0661	0.2316	19.6450/0.3856
SR3 [11]	109.3555	0.0209	0.1497	22.3090/0.5885
Manga109 [50] Dataset				
FDEDiff(ours)	**174.1602**	**0.0888**	**0.2282**	**14.4290/ 0.3285**
RCAN [4]	252.2228	0.1642	0.3085	15.4520/0.3699
SwinIR [8]	247.3774	0.1513	0.3058	15.4350/0.3744
ESRGAN [6]	244.6047	0.2030	0.3177	13.8800/0.2334
SR3 [11]	204.9557	0.1002	0.2927	14.3390/0.3449
Set5 [51] Dataset				
FDEDiff(ours)	**270.8795**	**0.0715**	**0.2319**	**18.7340/0.5511**
RCAN [4]	332.3032	0.0902	0.2860	21.2820/0.6002
SwinIR [8]	309.7468	0.0985	0.2721	21.5020/0.6149
ESRGAN [6]	373.4053	0.1451	0.3209	15.6140/0.2808
SR3 [11]	373.4053	0.1451	0.3209	15.6140/0.2808

Note: ↑ means the higher the value, the better. ↓ means the lower the value, the better.

**Table 3:** Image quality evaluation from $64^{2}\to 256^{2}$.

Method	FID↓	KID↓	LPIPS↓	PSNR↑/ SSIM↑
Thangka Dataset				
FDEDiff(ours)	**63.7272**	**0.0092**	**0.0815**	**27.3400/0.7339**
RCAN [4]	120.9347	0.0497	0.1088	29.8880/0.8027
SwinIR [8]	111.0603	0.0421	0.1073	30.0825/0.8060
ESRGAN [6]	64.8590	0.0019	0.1019	25.1050/0.6609
SR3 [11]	66.7570	0.0064	0.0921	25.6780/0.6433
Manga109 [50] Dataset				
FDEDiff(ours)	**80.6965**	**0.0151**	**0.1149**	**20.4740/0.6766**
RCAN [4]	82.7384	0.0164	0.1127	22.8300/0.7946
SwinIR [8]	81.3785	0.0161	0.1226	22.6070/0.7837
ESRGAN [6]	122.1136	0.0399	0.1799	18.8000/0.5567
SR3 [11]	94.7013	0.0164	0.1211	18.7290/0.5537
Set5 [51] Dataset				
FDEDiff(ours)	**61.2103**	**0.0830**	**0.0797**	**27.5990/0.7995**
RCAN [4]	71.6372	0.0983	0.0846	30.5980/0.8776
SwinIR [8]	62.4883	0.0842	0.0834	30.2430/0.8723
ESRGAN [6]	91.1225	0.0912	0.1339	24.0600/0.6624
SR3 [11]	67.6148	0.0847	0.1293	24.8740/0.6701

Note: ↑ means the higher the value, the better. ↓ means the lower the value, the better.

**Table 4:** Image quality evaluation from $128^{2}\to 512^{2}$.

Method	FID↓	KID↓	LPIPS↓	PSNR↑/ SSIM↑
Thangka Dataset				
FDEDiff(ours)	**42.7768**	**0.0024**	**0.0925**	**27.1530/0.6699**
RCAN [4]	90.6773	0.0195	0.2101	32.1599/0.8133
SwinIR [8]	90.8254	0.0198	0.2108	31.9854/0.8087
ESRGAN [6]	44.2777	0.0029	0.1088	25.6269/0.6403
SR3 [11]	44.9661	0.0019	0.1170	24.7120/0.5726
Manga109 [50] Dataset				
FDEDiff(ours)	**60.3558**	**0.0116**	**0.1683**	**22.8990/0.7455**
RCAN [4]	61.5298	0.0201	0.1720	24.3040/0.8074
SwinIR [8]	62.3904	0.0171	0.1796	24.3840/0.8104
ESRGAN [6]	64.2940	0.0792	0.1798	21.2390/0.6838
SR3 [11]	62.0121	0.0159	0.1770	20.3920/0.5717
Set5 [51] Dataset				
FDEDiff(ours)	**27.3981**	**0.0938**	**0.1244**	**29.6260/0.7689**
RCAN [4]	29.1625	0.0945	0.1396	31.7850/0.8991
SwinIR [8]	28.5705	0.0947	0.1376	32.0440/0.8903
ESRGAN [6]	29.7088	0.0991	0.1878	26.9080/0.7935
SR3 [11]	30.4452	0.0968	0.1519	28.2400/0.6904

Note: ↑ means the higher the value, the better. ↓ means the lower the value, the better.

### Ablation study

**Fig 10:**
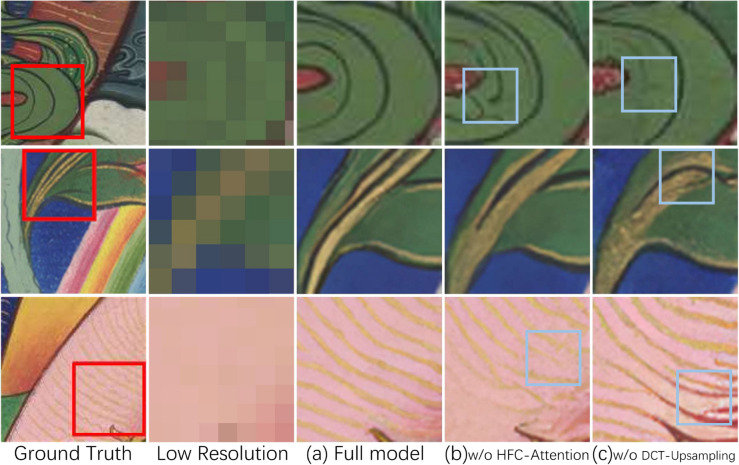
Qualitative evaluation results of ablation study. The proposed HFC-Attention and DCT-Upsampling allow our model to produce visually more accurate results in lines and textures. In contrast, the fourth column (without HFC-Attention) shows incorrect high-frequency details and smoother results due to the model’s insensitivity to high-frequency information. The fifth column (without DCT-Upsampling) shows missing lines because the model cannot correctly identify line semantics.

We conducted a series of ablation experiments to demonstrate the effectiveness of our model. Fig 10 shows the experimental results. To verify the effectiveness of HFC-Attention, we replaced HFC-Attention with Self-Attention. As can be seen in the rectangular boxes in column (b) of Fig 10, the high-frequency information in some areas is lost. This is because the model without HFC-Attention does not effectively focus on high-frequency information, resulting in the model’s inability to fully learn high-frequency information. It is noteworthy that after removing HFC-Attention from our model, LPIPS deteriorated from 0.1467 to 0.1544, while SSIM only decreased from 0.5958 to 0.5943. This indicates that the structural similarity of the results changed minimally, but perceptual quality showed significant degradation, which further demonstrates the improvement of HFC-Attention on high-frequency details.

To validate the effectiveness of DCT-Upsampling, we replaced DCT-Upsampling with nearest neighbor upsampling. In the rectangular boxes in column (c) of Fig 10, we can see that some lines are incomplete or have incorrect colors. This is because the model lacks DCT-Upsampling, which causes damage to the semantic information of some remaining lines during the upsampling stage of U-Net.

Table 5 shows the quantitative evaluation of the ablation experiments. It is evident that the absence of HFC-Attention and DCT-Upsampling in the model leads to varying degrees of decline in most metrics.

**Table 5:** Image quality evaluation of the ablation study.

Method	PSNR↑	SSIM↑	FID↓	KID↓	LPIPS↓
FULL	22.4200	0.5958	108.7645	0.0194	0.1467
w/o HFC-Attention	22.6740	0.5943	106.4146	0.0196	0.1544
w/o DCT-Upsampling	21.9640	0.5546	108.8706	0.0208	0.1525

Note: ↑ means the higher the value, the better. ↓ means the lower the value, the better.

To enable a fair comparison between DCT-based and spatial domain upsampling methods within identical architectures, we conducted comparative experiments using three super-resolution models: Real-ESRGAN [7], CAL_GAN [52], and Swin2SR [53]. While maintaining the principal structure of each model, we solely replaced their original spatial domain upsampling modules with the proposed DCT-Upsampling approach. All models were trained on the Thangka dataset using identical training strategies and subsequently evaluated on the Thangka test set. As shown in Table 6, the quantitative evaluation demonstrates consistent improvements in both PSNR and SSIM metrics for the DCT-Upsampling variants (with average gains of 0.0445 dB in PSNR and 0.003 in SSIM). These results indicate that the DCT-domain upsampling approach achieves superior effectiveness compared to spatial domain methods when implemented within the same architectural framework.

**Table 6:** Quantitative improvement of DCT-upsampling over spatial domain methods in SISR models.

Method		PSNR↑	SSIM↑
Real-ESRGAN [7]	Original	26.7416	0.7352
	DCT-Upsampling	26.7644	0.7373
CAL_GAN [52]	Original	29.0828	0.7908
	DCT-Upsampling	29.1701	0.7972
Swin2SR [53]	Original	30.2656	0.7714
	DCT-Upsampling	30.2891	0.7719

Note: ↑ means the higher the value, the better.

### Limitations

While our method demonstrates promising results in Thangka image super-resolution, several limitations warrant further consideration. First, occasional color deviations persist in generated images. This phenomenon stems from the spectral bias of Group Normalization layers in the DCTU-Net architecture, which may disproportionately suppress chromatic signals during iterative denoising.

Second, the current Thangka dataset, though large in scale (82,688 pairs), exhibits inherent constraints in diversity. It primarily focuses on common artistic styles and may not fully represent rare Thangka subtypes (e.g., Hei Tang [black Thangka]) or historical variations across different schools (e.g., Miantang vs. Qinzi traditions). Additionally, the synthetic degradation process used to generate low-resolution images—idealized bicubic downsampling—diverges from real-world Thangka deterioration patterns, which often involve complex mixed artifacts such as stains, creases, or partial pigment loss. The robustness of the model in real degraded scenarios still needs to be verified.

Finally, computational demands present barriers. The diffusion-based framework requires extensive training iterations and struggles with high memory consumption during high-resolution generation (e.g., 512×512). These factors hinder deployment on resource-constrained devices.

These limitations, however, do not diminish the methodological advancements but rather outline targeted directions for future work, such as hybrid degradation modeling and lightweight frequency-domain operators.

## Conclusion

This study proposes a frequency domain enhanced diffusion model framework (FDEDiff) to solve unique challenges in super-resolution of Thangka images. The proposed method has achieved significant advantages in both qualitative and quantitative evaluation. FDEDiff proves how AI-driven restoration technology fits with the ethics of cultural heritage protection - maintaining the original characteristics of art and culture while restoring it. This paradigm opens up new paths for the application of frequency domain diffusion models to other cultural heritages with similar structures. Future work will focus on efficiency optimization (combined with potential diffusion architecture), cross-cultural generalization (optimizing models for different types of cultural heritage) and hybrid degradation modeling.
